# P-479. Mortality Risk Factors in ECMO Pediatric Patients with Cannulation due to Infection: a 15-Year Tertiary Health Care Center Experience

**DOI:** 10.1093/ofid/ofaf695.694

**Published:** 2026-01-11

**Authors:** Caitlyn L Margol, Ali Abolhassani, Muhammad Ashraf, Alleigh Wettstein, Shawn Doss, Luke Guy, Grace Thayer, Karim Jandali, Malek Moumne, Pinkalkumar Patel, Laura L Hampton, Ingrid Camelo

**Affiliations:** Medical College of Georgia at Augusta University, Atlanta , GA; Medical College of Georgia at Augusta University, Atlanta , GA; Wellstar Medical College of Georgia Health Medical Center, Augusta, Georgia; Medical College of Georgia at Augusta University, Atlanta , GA; Medical College of Georgia at Augusta University, Atlanta , GA; Medical College of Georgia at Augusta University, Atlanta , GA; Medical College of Georgia at Augusta University, Atlanta , GA; Medical College of Georgia at Augusta University, Atlanta , GA; Medical College of Georgia at Augusta University, Atlanta , GA; Wellstar Medical College of Georgia Health Medical Center, Augusta, Georgia; Medical College of Georgia at Augusta University, Atlanta , GA; Children's Hospital of Georgia, Augusta, Georgia

## Abstract

**Background:**

Extracorporeal membrane oxygenation (ECMO) provides intervention for critically ill pediatric patients due to severe infection. This project evaluates whether pediatric patients cannulated for ECMO due to infection have higher odds of mortality than those cannulated for other pathologies without infection at the time of cannulation.

**Table 1:**
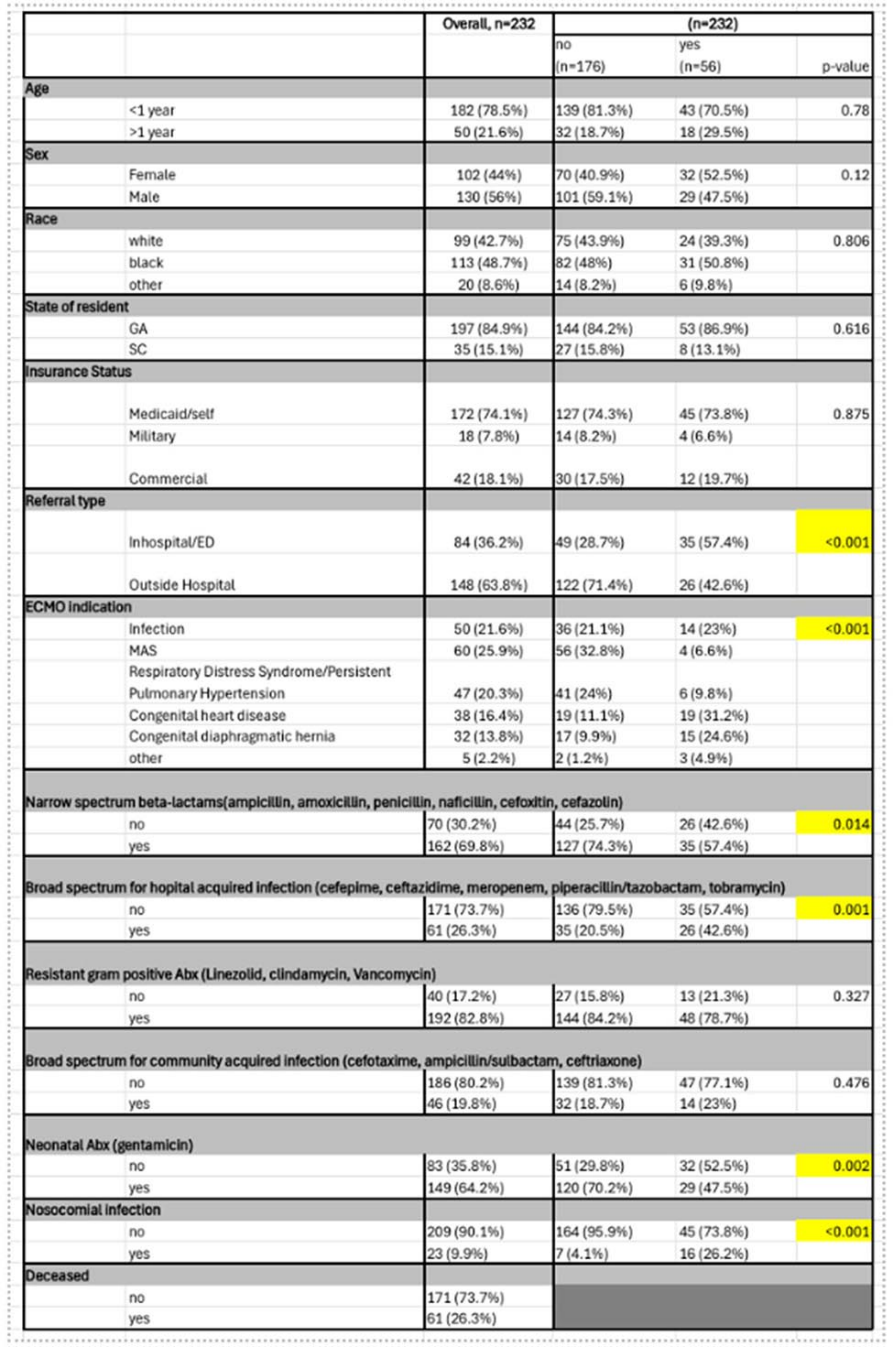
Descriptive Analysis of Patient Demographics

**Table 2:**
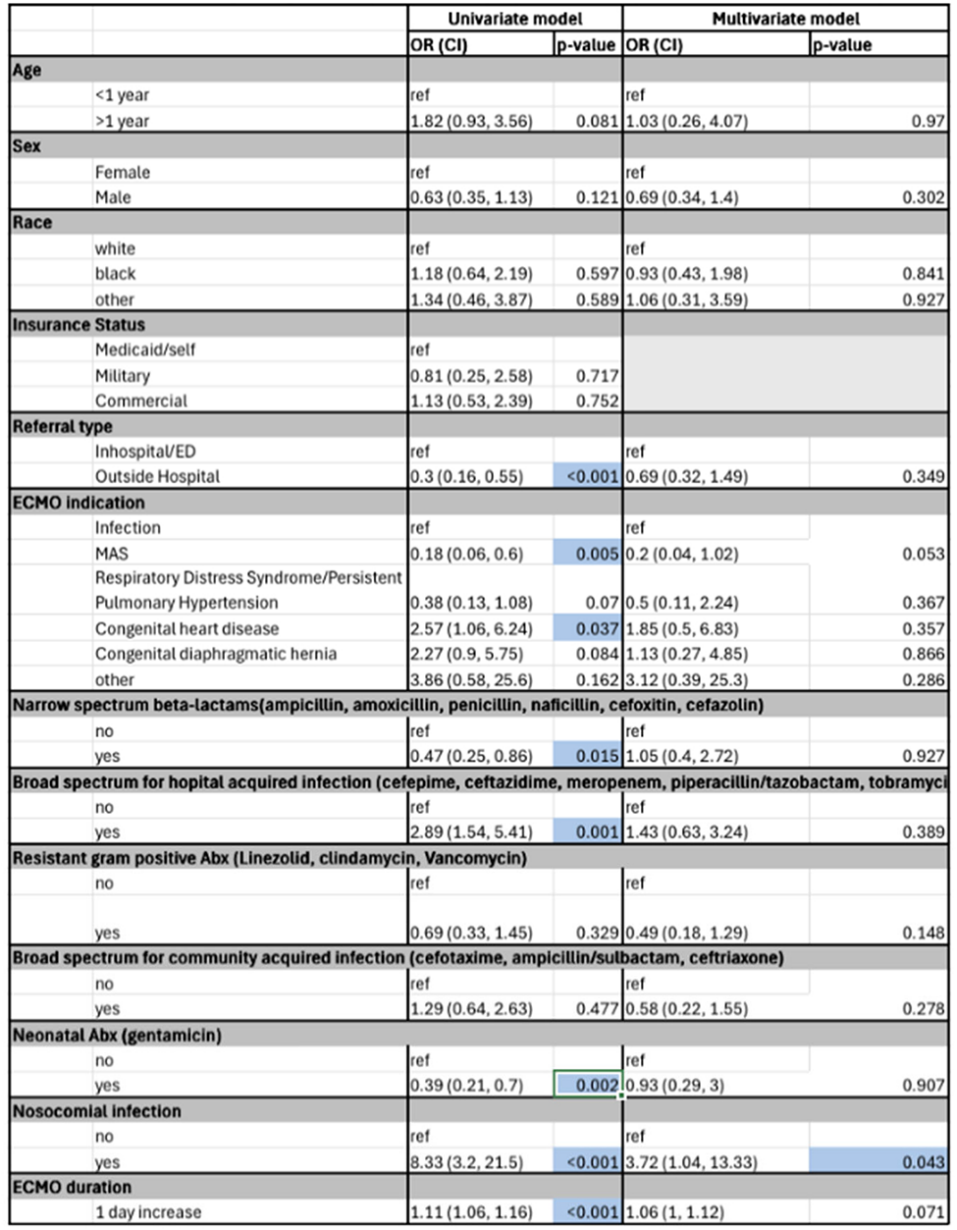
Univariate and Multivariate Logistic Regression to Predict Mortality in Pediatric ECMO Patients (N=232)

**Methods:**

Retrospective cohort study of 232 pediatric patients undergoing ECMO therapy. Indications for ECMO included infection, congenital diaphragmatic hernia (CDH), congenital heart disease (CHD), respiratory distress syndrome/persistent pulmonary hypertension (RDS/PPHN), and meconium aspiration syndrome (MAS). Descriptive statistics were used to summarize patient characteristics. Univariate logistic regression models evaluated the association between mortality, demographics, and ECMO indication. The multivariate model included age, sex, race, indication for ECMO, exposure to antibiotics categories defined by National Healthcare Safety Network (NHSN) criteria, and nosocomial infection status.

**Results:**

Of 232 patients, 50 (21.6%) were cannulated due to infection. In the univariate model, patients with MAS had significantly lower odds of mortality (OR=0.18, (95% CI 0.06-0.6) p=0.005) and patients with CDH had significantly higher odds of mortality (OR=2.57, (95% CI 1.06-2.64) p=0.003) compared to patients with infection as indication for cannulation. Patients on broad-spectrum antimicrobials used for hospital-acquired infections had higher odds of mortality (OR=2.89, (95% CI 1.5-5.4) p=0.001), and those exposed to narrow-spectrum antimicrobials had lower odds of mortality (OR=0.47, (95% CI 0.3-0.9) p=0.015). In the multivariate analysis, patients with nosocomial infections had 3.7 increased odds of mortality (OR=3.72, (95% CI 1-13) p=0.043).

**Conclusion:**

While no significant difference in odds of increased mortality was noted between indication groups in multivariate model, nosocomial infection during ECMO was the only independent predictor of mortality with nearly a 4-fold increase. Continued surveillance strategies, judicious use of antimicrobials and prevention for nosocomial infections should be prioritized towards improving survival in this population.

**Disclosures:**

All Authors: No reported disclosures

